# Great Bolgar’s historical genetics: a genomic study of individuals from burials close to the Greek Chamber in the 14th century

**DOI:** 10.18699/vjgb-25-45

**Published:** 2025-06

**Authors:** T.V. Andreeva, A.D. Soshkina, S.S. Kunizheva, A.D. Manakhov, D.V. Pezhemsky, E.I. Rogaev

**Affiliations:** Research Centre for Genetics and Life Sciences, Sirius University of Science and Technology, Sirius Federal Territory, Krasnodar region, Russia Centre of Genetics and Genetic Technologies, Lomonosov Moscow State University, Moscow, Russia Vavilov Institute of General Genetics of the Russian Academy of Sciences, Moscow, Russia; Vavilov Institute of General Genetics of the Russian Academy of Sciences, Moscow, Russia; Research Centre for Genetics and Life Sciences, Sirius University of Science and Technology, Sirius Federal Territory, Krasnodar region, Russia Vavilov Institute of General Genetics of the Russian Academy of Sciences, Moscow, Russia; Research Centre for Genetics and Life Sciences, Sirius University of Science and Technology, Sirius Federal Territory, Krasnodar region, Russia Vavilov Institute of General Genetics of the Russian Academy of Sciences, Moscow, Russia; Research Institute and Museum of Anthropology, Lomonosov Moscow State University, Moscow, Russia; Research Centre for Genetics and Life Sciences, Sirius University of Science and Technology, Sirius Federal Territory, Krasnodar region, Russia Department of Psychiatry, UMass Chan Medical School, Shrewsburry, MA, USA

**Keywords:** ancient DNA, genome, massive parallel sequencing, paleoanthropology, Bolgar, Greek ChamberFor, древняя ДНК, геном, параллельное секвенирование, палеоантропология, Болгар, Греческая палата

## Abstract

Bolgar was one of the most significant mediaeval cities in Eastern Europe. Before the Mongol conquest, it served as a major administrative centre of Volga Bulgaria, and after 1236, it temporarily functioned as the capital of the Golden Horde. Historical, archaeological, and paleoanthropological evidence indicates a mixed population of this city during the 13th–15th centuries; however, the contributions of exact ethnic groups into its genetic structure remain unclear. To date, there are no genetic data for this medieval group. For the first time, using massive parallel sequencing methods, we determined whole-genome sequences for three individuals from Bolgar who were buried in the early 14th century close to the so-called “Greek Chamber”. The average coverage of the studied genomes ranged from x0.5 to x1.5. We identified the genetic sex of the people (two men and one woman), and performed a population genetic analysis. The authenticity of the DNA studied and the low level of contamination were confirmed, and the mitochondrial DNA haplogroups of all three individuals as well as the Y-chromosome haplogroups of two male individuals were determined. We used more than 2.7 thousand DNA samples from representatives of ancient and modern populations that had been previously published to perform a comparative population-genetic analysis. Whole-genome data analysis employing uniparental markers (mitochondrial DNA and Y chromosome) and autosomal markers revealed genetic heterogeneity in this population. Based on PCA and f4- statistics analysis, a genetic connection was identified between one of the individuals (female) and modern Finno-Ugric peoples of the Volga-Ural region. Genomic analysis of the other two individuals suggests their Armenian origin and indicates migrant influx from the Caucasus or Anatolia. The results align well with archaeological and paleoanthropological findings and significantly enhance them by reconstructing the contributions of the indigenous population to the formation of the mediaeval Bolgar population structure.

## Abstract (ru)

Великий Болгар – один самых значительных средневековых городов Восточной Европы, до монгольского завоевания был крупным административным центром Волжской Булгарии, а после 1236 г. некоторое время выполнял функции столицы Золотой Орды. Исторические, археологические и палеоантропологи ческие данные свидетельствуют о смешанном составе населения этого города в XIII–XV вв., однако вклад тех или иных этнических групп в его генетическую структуру остается не вполне ясным, и до сих пор отсутствуют генетические данные для этой группы средневекового населения. Мы впервые с применением методов масштабного параллельного секвенирования определили полногеномные последовательности у трех жителей Великого Болгара, погребенных в первой половине XIV в. у так называемой Греческой палаты. Среднее покрытие исследованных геномов составило от х0.5 до х1.5, что позволило определить генетический пол индивидов (двое мужчин и одна женщина) и провести популяционно-генетический анализ. Были подтверждены аутентичность исследованной ДНК и низкий уровень контаминации, а также определены гаплогруппы митохондриаль- ной ДНК всех трех индивидов и гаплогруппы Y-хромосомы двух индивидов мужского пола. Для проведения сравнительного популяционно-генетического анализа нами задействованы опубликованные ранее данные геномного секвенирования более 2.7 тыс. образцов ДНК представителей современных и древних популяций. Анализ полногеномных данных трех индивидов из Великого Болгара с использованием однородительских маркеров (митохондриальной ДНК и Y-хромосомы) и аутосомных маркеров показал генетическую гетерогенность исследованной группы населения. По результатам геномного анализа с применением аутосомных маркеров и метода главных компонент (PCA) и f4-статистики нами выявлена генетическая связь одного индивида (женщины) с современными финно-угорскими народами Волго-Уральского региона. Геномный анализ двух других индивидов предполагает их армянское происхождение и свидетельствует о существовании потока мигрантов с территории Кавказа или Анатолии. Полученные результаты хорошо согласуются с данными археологии и палеоантропологии, а также существенно дополняют их в части реконструкции вклада автохтонного населения в формирование популяционной структуры средневековых жителей Болгара.

## Introduction

Great Bolgar was a significant administrative center of two sequential medieval states in Eastern Europe, and one of the key cities of Volga Bulgaria, where Islam was adopted in 922. After the Mongol invasion and devastation in 1236, it was restored as the first capital of the Golden Horde. Volga Bulgaria was a polyethnic polity where Finno-Ugric peoples, Slavs, and the Turkic tribes (Bulgars) – who migrated to the interfluve of the Kama and the Great Cheremshan from the Azov region and the territory of Krasnodar Krai –coexisted. As part of the Golden Horde, the Volga region population maintained its complex ethnic composition, which was particularly characteristic of major urban centers such as Bolgar.

In its history and economy, due to its advantageous location on the main waterway of the East European plain – both during the Bulgar period and especially during the Golden Horde period – considerable importance was attributed to craft production and transcontinental trade. This circumstance contributed to the formation of a polyethnic urban population, as a significant portion of its inhabitants consisted of foreign merchants (Smirnov, 1951, 1972, 1974; Gening, 1989; Khalikov, 1989; Iskhakov, Izmailov, 2000; Bulgarica, 2012; Sitdikov, Bocharov, 2024).

Archaeological data as well as paleoanthropological materials indicate a mixed population composition in Great Bolgar throughout all stages of its existence (Trofimova, 1956; Postnikova, 1970, 1973; Efimova, 1991; Gazimzyanov, 2000, 2015). The contribution of steppe Turkic and indigenous Finno-Ugric populations to the anthropological makeup of its inhabitants is considered proven. The question of the contribution of the Azov Bulgars, who migrated to the Volga region in the early Middle Ages, as well as the participation of Slavic groups, remains a topic of discussion. During the Golden Horde period, new components that increased the ethnic diversity of the medieval Bolgar population emerged. A striking example of this is a series of skulls from burial sites at the “Greek Chamber”, which were identified by T.A. Trofimova as “Armenoid” upon initial study (Trofimova, 1949).

Although the population of Great Bolgar was predominantly Muslim, as indicated by archaeological data, there were also diasporas of other faiths present. For instance, the burial ground at Babiy Bugor is interpreted by several researchers as a Christian cemetery. However, the most vivid illustration of this is a site known as the “Greek Chamber”, which was a rectangular stone structure measuring 12.6 by 16.4 meters, oriented along the West-East axis. Still well visible at the beginning of the 18th century, it was scientifically documented for the first time during that period. Archaeological excavations of the “Greek Chamber” were carried twice: once in 1916 by V.F. Smolin and again in the mid-1940s by A.P. Smirnov. The structural and dimensional characteristics of the building itself, along with the gravestones featuring inscriptions in Armenian, described in a timely manner, allowed it to be identified as a small temple, similar to the construction from 1339 in Noravank (Armenia) or Armenian churches from the 14th century in Feodosia and Old Crimea (Smirnov, 1951). Temples of this type were typically twostoried, with the lower level serving as a burial chamber. The deciphered and repeatedly published epitaphs date from 1308 to 1335. This enabled A.P. Smirnov to reasonably assert that a necropolis of the Armenian colony, formed around a commemorative temple, had been discovered in Bolgar (Smirnov, 1951, 1958).

Further extensive paleoanthropological research in Bolgar has shown that the population buried at the “Greek Chamber” finds considerable analogies in the multidimensional craniometric space with groups interred in various Muslim mausoleums of the city, as well as near the Small Minaret, at the soil cemeteries of Excavations 45 and 191 (Gazimzyanov, 2000, 2015). This emphasizes the importance of employing paleogenetic methods as well as new paleoanthropological analyses

While genomic methods are already widely employed to study ancient peoples, populations, and individuals, genetic data related to the population of Great Bolgar are still lacking. Such information would greatly improve our understanding of its ethnic makeup provided by archaeological and paleoanthropological studies. A particular goal is to assess the contribution of the medieval population to the formation of the present-day peoples of the Volga region. In this study, for the first time, we have applied genomic analysis methods to investigate individuals buried in the territory of Great Bolgar

## Materials and methods

Paleoanthropological material (inventory numbers 8964, 8973, 8977) from the collections of the Research Institute and the Museum of Anthropology named after D.N. Anuchin at Moscow State University was used. The remains originate from burials dating to the first half of the 14th century, which were archaeologically examined by a Joint expedition of the Institute of Material Culture History and the Museum of the Tatar ASSR, led by A.P. Smirnov, in 1945 and 1947 in Bolgar, at the so-called “Greek Chamber”. This site was located approximately 150 meters west of the city wall, in an area currently occupied by the Bolgar grain terminal, and is not discernible on the surface today

Excavations of the ruins of the “Greek Chamber” and the surrounding territory to the south and southeast allowed A.P. Smirnov and A.M. Efimova to identify 113 Christian burials. From an archaeological perspective, these burials are characterized as “homogeneous/uniform”, contained within wooden coffins (the wood was found to be decayed, with numerous iron nails discovered), which were placed in rectangular pits measuring 2 × 0.8 meters, with rounded corners, vertical walls, and flat bottoms. The remains were found in an extended position on their backs, with heads facing west, faces upward, hands folded on their chests, and accompanied by a small number of personal items. However, it is noteworthy that some burials with gravestones and their fragments, as well as burials containing rich silk textiles embroidered with gold and silver threads, were also recorded; additionally, a temporal gold ring was discovered. A.P. Smirnov believed that his excavations uncovered a significant portion of this necropolis, estimating that it likely contained no more than 150 interments. Interestingly, while characterizing female ornaments, particularly temporal rings, A.P. Smirnov found analogies for some of them among the Slavic burial mounds of the Smolensk and Tver regions, attributed others to Bulgar prototypes from the 10th to 12th centuries, and associated some with artifacts from burials dated to the 12th to 14th centuries found in the Northern Caucasus (Smirnov, 1951).

Currently, considering our understanding of the multicomponent nature of Bulgar material culture, which is predominantly urban and where many elements lose their “ethno-defining” characteristics upon contact, one might question the significance of these observations. Nevertheless, based on paleoanthropology and paleogenetics, they have the potential to significantly enhance our understanding of the ethnic composition of the medieval population of Bolgar.

In 1948, the collection of skulls from the burials at the “Greek Chamber” was transferred to the Museum of Anthropology at Moscow State University. These specimens were first measured and reported by T.A. Trofimova, and subsequently re-examined by M.M. Gerasimova and published later by I.R. Gazimzyanov (Gazimzyanov, 2000; Trofimova, 1956).

Genetic analysis was performed using fragments of teeth from three individuals with the best-preserved anthropological material. Genomic DNA was extracted from fragments weighing 100–200 mg in clean rooms, where studies of modern materials had not been conducted, using a previously published method (Andreeva et al., 2022). The DNA extract and blank control were tested using the High Sensitivity DNA reagent kit (Agilent) on a Bioanalyzer 2100 (Agilent). Fragmented genomic libraries were prepared according to a single-stranded DNA-based protocol (Gansauge et al., 2017) and sequenced firstly on Illumina MiSeq (paired-end reads mode of 76+76 cycles) and then on Illumina NovaSeq 6000 in single-end read mode of 56 cycles.

We used AdapterRemoval v2 (Schubert et al., 2016) for trimming the adapter sequences of the raw reads. Short reads were mapped using the BWA program (Li, Durbin, 2009) with parameters adapted for short fragments of ancient DNA (Schubert et al., 2012) to the human reference genome (hg19/GRCh37 assembly), and to the mtDNA Cambridge Reference Sequence (NC_012920.1). The authenticity of the ancient DNA was evaluated using the MapDamage2 program (Jónsson et al., 2013). To determine the genetic sex of individuals, we calculated the ratio of reads mapped to the X and Y chromosomes to reads mapped to autosomes; reads with a mapping quality (MQ) greater than 30 were used for the assessment.

We used contamMix (Fu et al., 2013) to estimate the contamination of samples by mtDNA heterozygosity. For male individuals, the contamination level was additionally estimated by X-chromosome heterozygosity (Rasmussen et al., 2011).

Mitochondrial haplogroup was determined using Haplogrep 2 (Weissensteiner et al., 2016). The Y-chromosome haplogroup was determined using the Yhaplo program (Poznik, 2016). Then, in order to clarify the Y-chromosomal lineage, the markers of the corresponding haplogroup and all its derived branches were visually checked using the IGV browser (Robinson et al., 2011). Both Y-chromosomal haplogroup markers presented in the ISOGG database (version 15.73) (International Society…, 2020) and markers from the Yfull database (YFull, 2024) were used. To conduct a phylogeographic analysis of mtDNA using the BLAST service, we selected all mtDNA sequences close to the sequences of the studied samples (identity >99.98 %), as well as samples from the YFull and AmtDB databases (Ehler et al., 2019) belonging to the identified haplogroup, and used them to construct a phylogenetic tree using the mtPhyl program (Eltsov, Volodko, 2016).

For the analysis of genomic markers, pseudohaploid genotypes corresponding to the AADAR panel (version v54.1.p1), which includes 1,240 thousand genetic markers (Mallick et al., 2024), were obtained using the PileupCaller program with the “--randomHaploid” parameter. Population analysis was performed using principal component analysis (PCA). For this purpose, the genotypes of the studied samples from the burials at the “Greek Chamber” were projected onto the genetic variability of 2,775 representatives of 80 present-day European and Caucasian populations from the Human Origin panel (Lazaridis et al., 2016). PCA analysis was performed using smartpca from the EIGENSOFT software package (Patterson et al., 2006). To assess genetic similarity with ancient populations, f4-statistics were calculated. Pseudohaploid genotypes for the panel of 1,240 thousand genetic markers for the populations included in the analysis were obtained from the AADR database (version v54.1.p1). For the calculation, the ADMIXTOOLS v.7.0.1 (Patterson et al., 2012) and admixr v.0.9.1 (Petr et al., 2019) software packages were used. The “inbreed=YES” parameter was applied in the calculation. The results were visualized using the R/4.2 package (R Core Team, 2021).

## Results

**Fig. 1.**
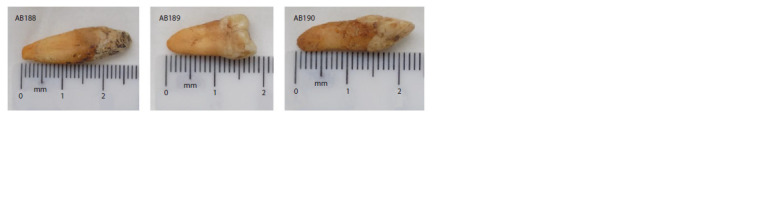
Anthropological material (teeth) used in the study.

**Fig. 2.**
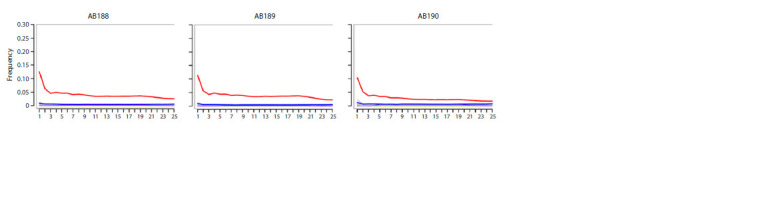
The profile of nucleotide substitutions for reads mapped to the reference mtDNA sequence, calculated with MapDamage2 (Jónsson et al., 2013). C>T transitions specific to ancient DNA are indicated by a red line. The X axis denotes the nucleotide position from the 5’ end of the DNA fragments.

Genomic DNA obtained from the teeth of three individuals (AB188, AB189, AB190) was used for genomic library preparation and sequencing (Table 1, Fig. 1). Bioinformatics analysis of the obtained short reads confirmed the authenticity of the DNA for each sample (Fig. 2). The proportion of reads mapped to the human reference genome ranged from 20 % to 66 %, indicating the high quality of the examined bone material and its suitability for whole-genome analysis (Table 2). According to the results of the assessment of the ratio of the average coverage of sex chromosomes to autosomes, it was observed that two samples (AB188 and AB190) belong to males, while one (AB189), to a female. The genetic sex of all samples coincided with their phenotypic sex, previously determined by biological and anthropological methods.

**Table 1.**
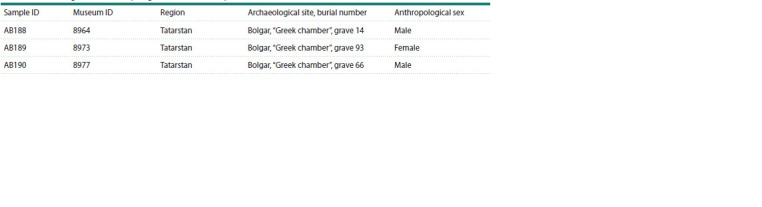
Archaeological and anthropological data of the samples

**Table 2.**
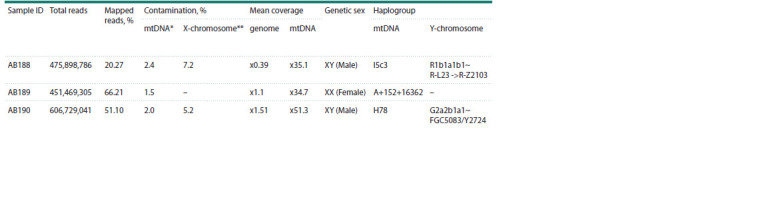
DNA sequencing statistics, mtDNA and Y-chromosome haplogroups * Based on contamMix (Fu et al., 2013). ** ANGSD was used to determine X-chromosome contamination in men (Korneliussen et al., 2014).

Based on the sequencing data, complete mitochondrial sequences of the studied individuals were determined, as well as their mitochondrial haplogroups. The mtDNA sequences of all three individuals belong to three different mitochondrial lineages (I5c3, A+152+16362, and H78); therefore, these individuals are not maternally related (Table 3).

**Table 3.**
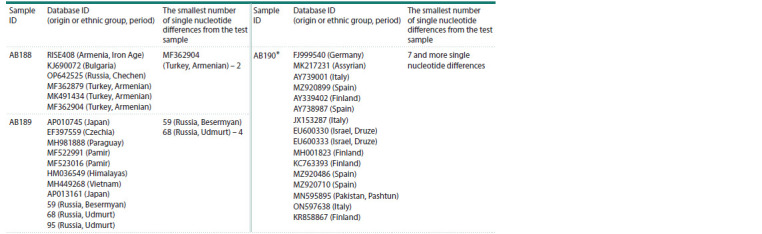
Variants of the mtDNA sequences found in the tested samples from the burials at the “Greek Chamber” * Several randomly selected mtDNA sequences from those selected by the percentage identity parameter >99.98 % are presented. The mtDNA sequences found are widely distributed across Europe and Western Asia.

For two male individuals, different Y-chromosome haplogroups were identified. The Y chromosome of AB188 belongs to haplogroup R1b1a1b1~ (R-Z2103). Individual AB190 is a carrier of haplogroup G2a2b1a1~ (FGC5083/Y2724). Therefore, the studied males are not related to each other through the paternal line.

**Fig. 3.**
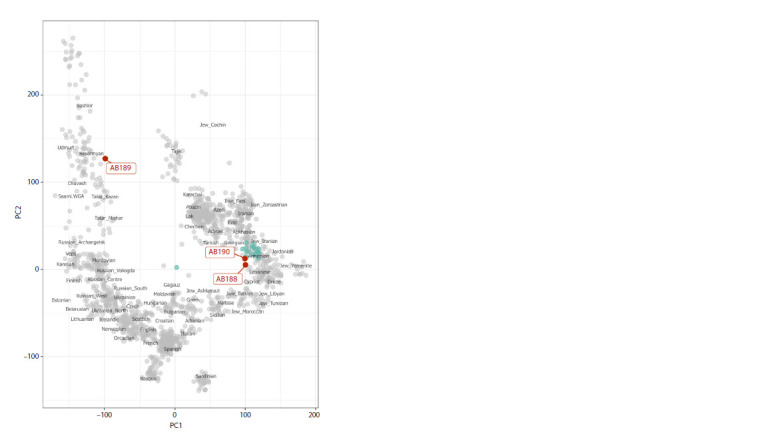
PCA plot visualizing Volga Bulgar samples (red dots) projected onto the first two (PC1 and PC2) components of genetic variability of present-day individuals from the Human Origin dataset (grey dots); present-day Armenians are marked with green dots.

A PCA indicated that both male samples (AB188 and AB190) cluster within present-day Caucasian populations, in close proximity to samples from present-day Armenia and Turkey. The female sample AB189 is projected near presentday populations of the Volga-Ural region (Fig. 3).

**Fig. 4.**
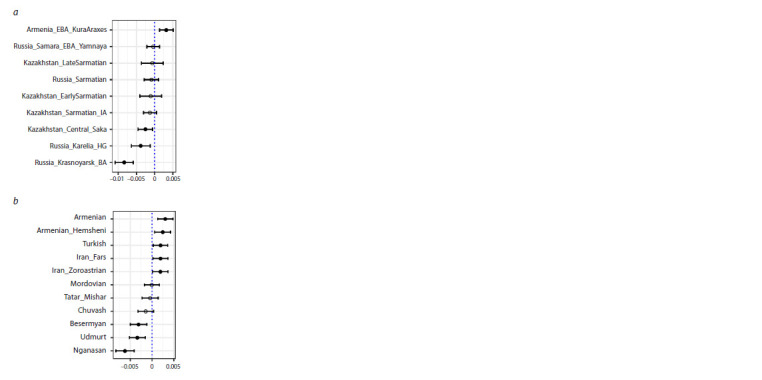
f4-statistics of the form of f4(Test, Mbuti; AB188 and AB190, AB189). Test – ancient (a) and present-day (b) populations.

For testing the potential genetic contribution and similarity to ancient populations, we performed a calculation of the f4-statistic of the form f4(Test, Mbuti; AB188 and AB190, AB189), where Test represents one of the tested ancestral populations, and the African Mbuti group was used as an outgroup. The results of the analysis (Tables 4, 5, Fig. 4) confirm a greater similarity of individual AB189 with ancient groups that formed the genetic substrate of the contemporary Finno-Ugric population (Russia_Karelia_HG and Russia_ Krasnoyarsk_BA) and with modern Siberian groups (the Besermyans, Udmurts, and Nganasans). In contrast, individuals AB188 and AB190 exhibit significantly more shared alleles with the population group from the Kura-Araxes culture of the Bronze Age in Armenia (Armenia_EBA_Kura_Araxes), which has made a substantial contribution to the genetic structure of contemporary Armenians, as well as with modern representatives of present-day Turks, and Iranians (Fig. 4).

**Table 4.**
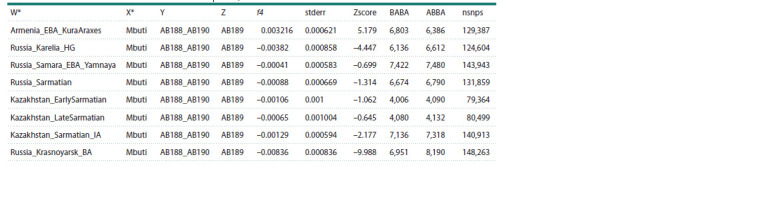
f4-statistics of the form f4(W,X;Y,Z) for the ancient samples from the AADR database Notе. Here and in Table 5: W – ancient samples from the AADR database; X – Mbuti; Y – АВ188 and АВ190; Z – АВ189; f4 – calculated f4-statistic; stderr – standard error; Zscore – calculated Z-score; BABA/ABBA – the number of ABBA/BABA patterns of allele sharing among tested populations; nsnps – allele number. * The labels for the ancient groups correspond to the AADR database (version v54.1.p1) of 1,240 K genetic markers.

**Table 5.**
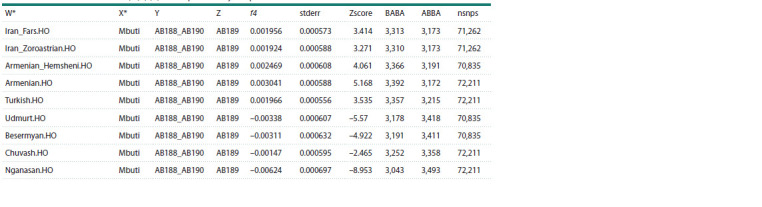
f4-statistics of the form f4(W,X;Y,Z) for the present-day samples from the AADR database * The labels for the population groups correspond to the Human Origin set of the AADR database (version v54.1.p1) with 600 K genetic markers.

## Discussion

Previous studies on Bolgar’s craniological series have shown that the local Finno-Ugric component is significantly represented in the population. Notably, the male skulls from the burials next to the “Greek Chamber” have frequently been likened to the skulls of present-day Armenians. In contrast, the female skulls exhibited closer alignment with the cranial characteristics typical of the local Finno-Ugric population (Trofimova, 1956; Efimova, 1991). The high-quality whole- genomic data we obtained from all three samples of individuals representing the medieval Bulgarian population enabled us to analyze both uniparental markers (mtDNA and the Y-chromosome) and autosomal markers, thereby providing insights into the probable origins of the studied individuals.

According to the population analysis utilizing autosomal genetic markers (Fig. 3), the two male samples and the female sample (AB189) from grave 93 next to the “Greek Chamber” differ significantly. According to the projection of the first two principal components, the female sample is most similar to modern-day Besermyans, who speak a Finno-Ugric language, and to Chuvash and Kazan Tatars, who speak Turkic languages. All these groups have a significant amount of autochthonous substrate in their genetic makeup. These modern communities are descendants of a Finno-Ugric people that lived in regions that were once part of Volga Bulgaria and the Golden Horde.

The maternal lineage (mtDNA) of individual AB189 belongs to the East Eurasian haplogroup A+152+16362. This mitochondrial haplogroup is distributed mainly in East Asia and among the indigenous population of America. Among the present-day European populations, haplogroup A is most prevalent in Tatars and Bashkirs of the Volga-Ural region, where it accounts for up to 3.6 % (Malyarchuk et al., 2010).

In the databases of present-day and ancient DNA (GenBank, AmtDB, Yfull), we searched for complete mitochondrial sequences that are most similar to the mtDNA sequence of sample AB189 (percent identity >99.94). The analysis of the phylogenetic tree constructed using the mtPhyl program revealed that sample AB189 has a substitution at position 93, which allows it to form a common clade with samples from modern Udmurts and Besermyans (Fig. 5). Thus, the results of the genetic analysis indicate that the woman we studied has a genetic profile similar to that of the contemporary Finno-Ugric population of the Volga-Ural region. Due to the current lack of genomic data for the medieval population of the Volga region, conducting a comparative analysis of the examined individuals with the ancient inhabitants of this area is challenging. Nevertheless, paleoanthropological data suggest similarities between the medieval and modern indigenous populations (Efimova, 1991), which allows us to hypothesize that the woman from burial 93 next to the “Greek Chamber” was a representative of the local Finno-Ugric tribes.

The two male samples are positioned on the PCA plot close to the present-day populations of the eastern Mediterranean (Turks, Jews, Libyans, and Cypriots) and the Caucasus (Georgians, Armenians), and they are quite far from the samples that belong to the Volga-Ural region’s populations (Fig. 3).

The mtDNA of male AB188 belongs to haplogroup I5c3. Sequences of contemporary mtDNA samples of this haplogroup, as represented in the GenBank database, have been identified exclusively among individuals from Caucasian populations, predominantly among Armenians. Notably, among ancient samples, haplogroup I5c, which is ancestral to haplogroup I5c3, was also found in an individual who lived in the territory of present-day Armenia and belonged to the Lchashen-Metsamor culture (1209–1009 BCE) (Allentoft et al., 2015). The Y chromosome of male AB188 belongs to haplogroup R1b1a1b1~ (R-Z2103). Due to insufficient genome coverage, we were unable to determine subsequent markers within this Y clade. This haplogroup is part of the larger Y-chromosomal clade R1b, which is widespread in modern populations of Western Europe. However, haplogroup R-Z2103 identified in individual AB188 belongs to the Eastern European lineage R1b-L23, which is most prevalent in the Caucasus, Turkey, and the Ural region, where its frequency reaches up to 10 % (Myres et al., 2011).

According to the YFull database, the largest number of contemporary representatives of haplogroup R-Z2103 originates from Armenia. Some estimates suggest that between 19 and 23 % of the modern population of Armenia belong to various branches of this haplogroup (FamilyTreeDNA; Hovhannisyan et al., 2025). The results of the genomic analysis of individual AB188 from burial 14, based on data obtained from autosomal markers as well as maternal and paternal lineage markers, indicate that the studied male from the burial site in the “Greek Chamber” in Volga Bulgaria had Armenian origins.

The maternal lineage of the second male individual (AB190) belongs to mitochondrial haplogroup H78. This mitochondrial haplogroup originated from an ancestral branch of haplogroup H due to a single substitution at position 7002. Phylogenetic analysis based on sequences available in databases that are similar to the mtDNA of individual AB190 (percent identity >99.93) revealed that only two sequences in the databases contain the same substitution (7002), both belonging to contemporary representatives of the Druze ethnic group residing in northern Israel (Fig. 5). However, it is important to note that the sequence of AB190 differs from the sequences of these two individuals by at least nine positions in the mtDNA, whereas the differences between mtDNA of AB190 and the sequences of the root haplogroup H, the most common haplogroup in Europe, as well as numerous sequences of sister lineages to H78, amount to only 7–8 substitutions. Thus, drawing conclusions about the likely geographical or ethnic origins of the maternal lineage of individual AB190 is challenging, although one might hypothesize the existence of a common ancient ancestor with the Druze of Israel.

The analysis of the male lineage of this man from Great Bulgaria showed that his Y-chromosomal haplogroup belongs to haplogroup G2a2b1a1~ (FGC5083/Y2724), which is predominantly found in modern populations of the Caucasus and is most widely distributed in contemporary Turkey. Recent data on the most likely origin of modern Armenian groups from ancient Anatolia (Hovhannisyan et al., 2025) indicate a high level of genetic similarity between Armenians and the inhabitants of Turkey. Thus, the second man we studied (AB190, burial 101) was also likely a migrant from the territory of Armenia or Anatolia.

## Conclusion

The data we obtained are in line with the historical and archaeological evidence regarding the existence of a segment of the population in Great Bulgaria that was represented by migrants or merchants of Armenian descent. Previously, based on craniological data, a hypothesis was proposed suggesting that Armenians who migrated to Bulgaria took local women as wives (Trofimova, 1956). The results of our analysis partially support the hypothesis of different origins of the men and women buried at the “Greek Chamber”. However, to provide evidence for such marital practices, it is necessary to increase the sample size and include potential descendants from such mixed marriages.

## Conflict of interest

The authors declare no conflict of interest.
